# Recurrent Pneumocystis Pneumonia with Uncommon Radiographic Presentation

**DOI:** 10.7759/cureus.2125

**Published:** 2018-01-29

**Authors:** Ayushi Dixit, Rayhan Shariff, Sherleen Gandham, Ravi Bhavsar, Jazila Mantis, Victoria Vapnyar

**Affiliations:** 1 Internal Medicine, Icahn School of Medicine, Mount Sinai / Queens Hospital Center, New York.; 2 Infectious Disease, Icahn School of Medicine, Mount Sinai / Queens Hospital Center, New York.

**Keywords:** pcp, pneumocystis pneumonia, hiv, opportunistic infection

## Abstract

Pneumocystis carinii pneumonia (PCP) is a common opportunistic infection of the pulmonary parenchyma seen in the immunocompromised host. The clinical presentation and radiographic findings are varied, with the latter ranging from normal to bilateral ground-glass opacities with cyst formation. We present a case of a 46-year-old woman with a history of human immunodeficiency virus (HIV) with multiple treated prior episodes of PCP, who was found to have an impressive presentation on high-resolution chest computed tomography (HRCT).

## Introduction

The incidence of Pneumocystis jirovecii pneumonia (PCP), a leading cause of opportunistic infection in human immunodeficiency virus (HIV) infected patients, has dramatically decreased due to effective antiretroviral therapy (ART) and the use of PCP prophylaxis [1]. The most common radiographic abnormalities in patients with PCP are diffuse, bilateral, interstitial, or alveolar infiltrates [2]. Here, we report an HIV infected patient with PCP presenting with extensive bilateral cystic lung disease.

## Case presentation

A 46-year-old woman with HIV disease diagnosed twenty-seven years earlier and active crack cocaine user presented with fever, cough, shortness of breath, hemoptysis, night sweats, and weight loss. The patient had prior episodes of PCP with the last episode two years earlier diagnosed by bronchoscopy at another facility. The patient was non-adherent to antiretroviral therapy and medical care and reported variable adherence to PCP prophylaxis with atovaquone. 

**Figure 1:**
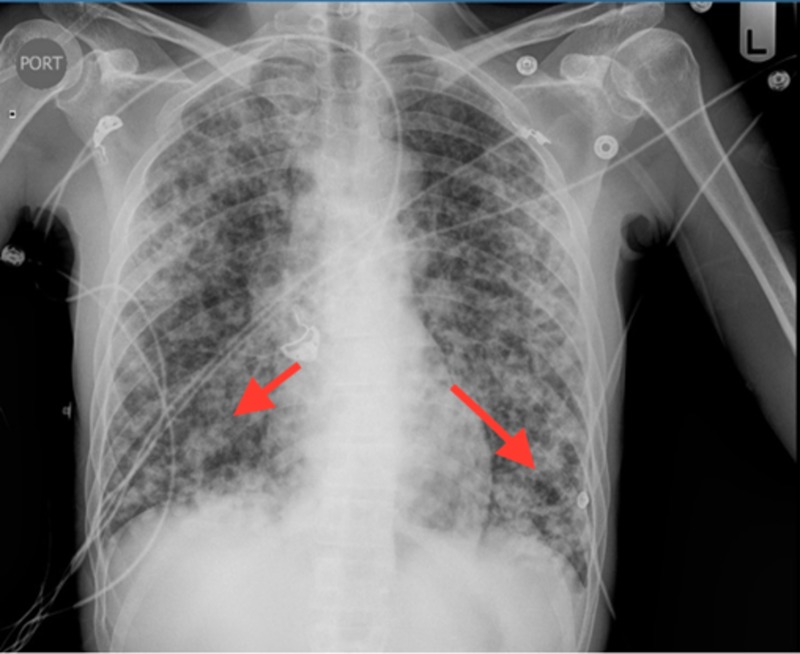
Chest x-ray revealing bilateral coarse reticulonodular opacities and cysts

**Figure 2:**
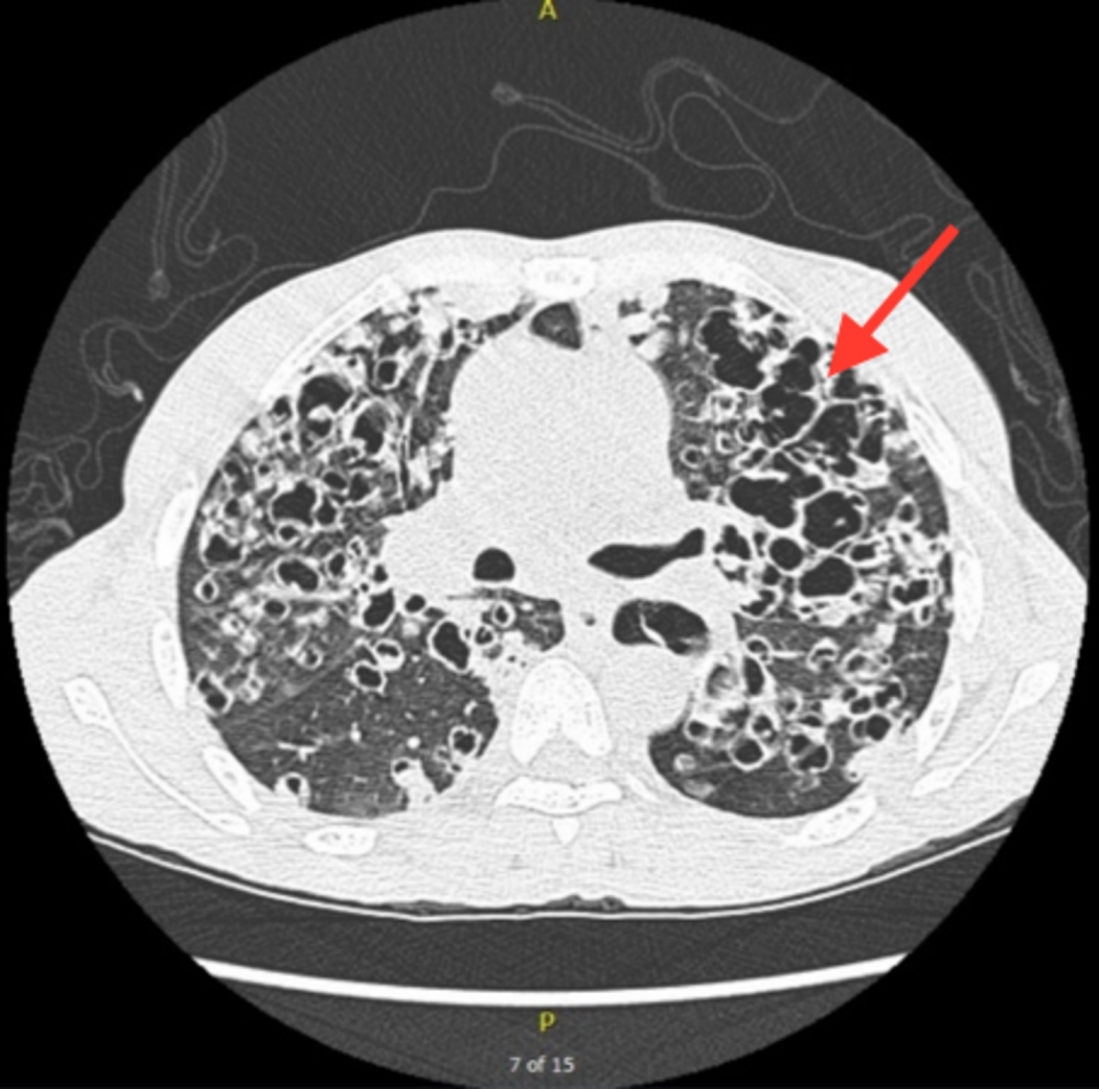
High-resolution computed tomography (CT) revealing multiple thick-walled cysts and nodules throughout bilateral lung fields

**Figure 3:**
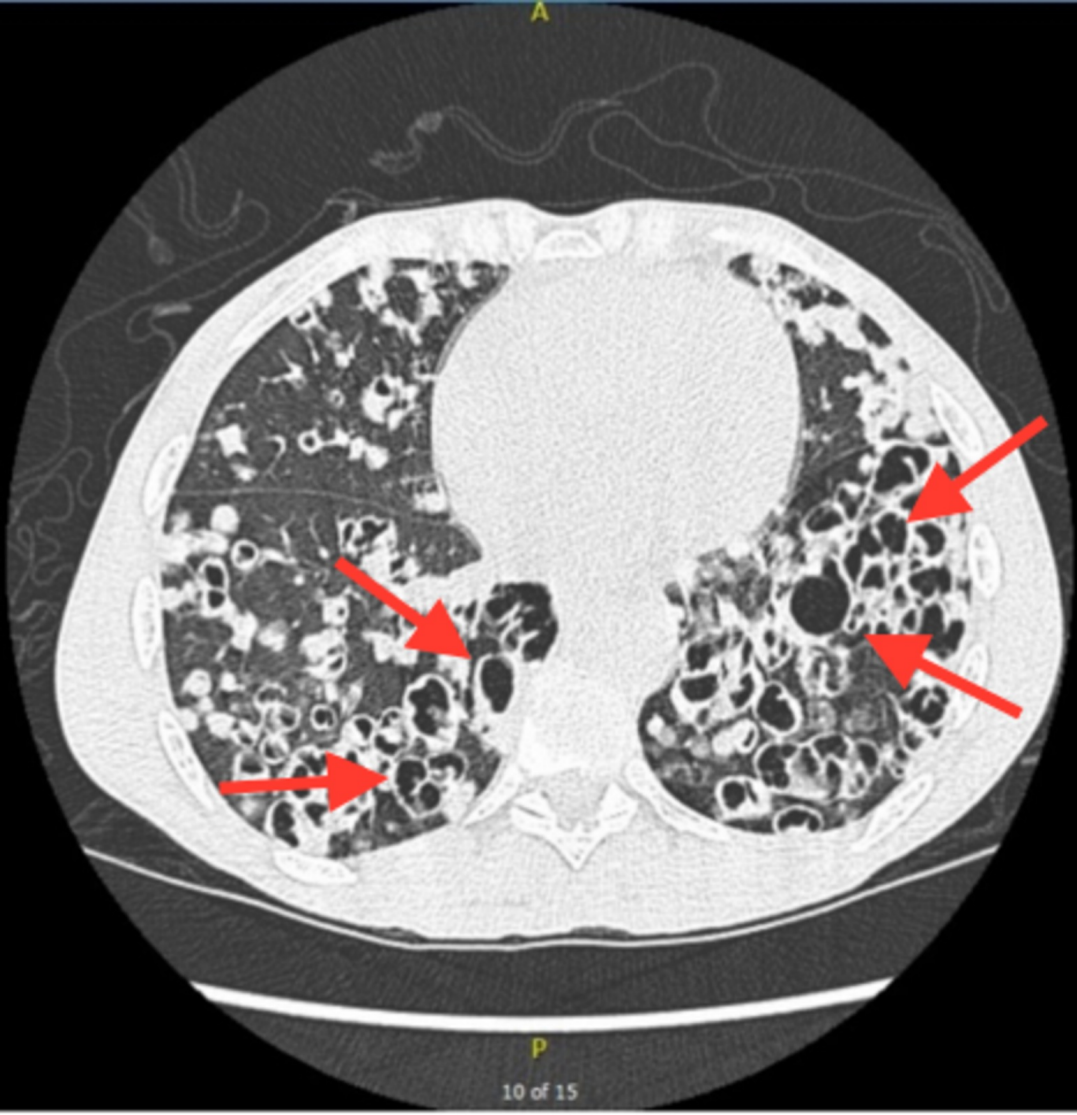
High-resolution computed tomography (CT) revealing multiple thick-walled cysts and nodules throughout bilateral lung fields

Chest x-ray revealed bilateral coarse reticulonodular opacities and cysts (Figure 1). High-resolution computed tomography (HRCT) revealed innumerable thick-walled cysts and nodules throughout bilateral lung fields (Figures 2, 3). Laboratory findings included CD4 34 (3%) and negative serum QuantiFERON®-TB (Qiagen, Hilden, Germany), serum Histoplasma antigen, and serum cryptococcal antigen. Three sputum specimens were negative for acid-fast bacilli and remained negative for TB culture. A bronchoscopy with bronchoalveolar lavage (BAL) was positive for PCP. The patient was treated with intravenous pentamidine due to a past history of sulfa allergy.

## Discussion

Even though the incidence of PCP in HIV infected patients in the US has declined from 29.9 per 1,000 person-years between 1994 - 1997 to 3.9 per 1,000 person-years between 2003-2007 due to effective ART and the use of PCP prophylaxis, PCP remains one of the leading causes of opportunistic infection in HIV infected individuals, especially in those who are not receiving care or are undiagnosed [3]. Chest x-rays are initially normal in up to one-fourth of patients with PCP and the most common radiographic abnormalities are diffuse, bilateral, interstitial, or alveolar infiltrates. Less common radiographic presentations include lobar or segmental infiltrates, cysts, nodules, pleural effusions, and pneumothorax [2]. 

The differential diagnosis in HIV patients with pulmonary disease includes tuberculosis, non-tuberculous mycobacteria, fungal infections (including Histoplasma and Cryptococcus), cytomegalovirus, and Kaposi’s sarcoma [4]. Our patient presented with extensive bilateral cystic lung disease due to significant tissue injury and necrosis from recurrent PCP due to non-adherence with PCP prophylaxis and ART.

## Conclusions

PCP remains a significant cause of morbidity and mortality in HIV infected individuals who are non-adherent to or have limited access to medical care. Extensive tissue damage from severe PCP or recurrent PCP can lead to extensive cystic lung disease with increased risk of pneumothorax and poor prognosis.
